# Supplementary material for: Annealing-Dependent Morphotropic Phase Boundary in the BiMg0.5Ti0.5O3–BiZn0.5Ti0.5O3 Perovskite System
Source: Materials (Basel). 2022 Oct 9;15(19):6998. doi: 10.3390/ma15196998 (PMC9570801; doi:10.3390/ma15196998)
Supplement: Supplementary file 1 [file materials-15-06998-s001.zip › materials-1942516-supplementary.pdf]

Supporting Information

Annealing-Dependent Morphotropic Phase Boundary in the $BiMg_{0.5}Ti_{0.5}O_3$–$BiZn_{0.5}Ti_{0.5}O_3$ Perovskite System

João Pedro V. Cardoso [1], Vladimir V. Shvartsman [2,*], Anatoli V. Pushkarev [3], Yuriy V. Radyush [3], Nikolai M. Olekhnovich [3], Dmitry D. Khalyavin [4], Erik Čižmár [5], Alexander Feher [5] and Andrei N. Salak [1,*]

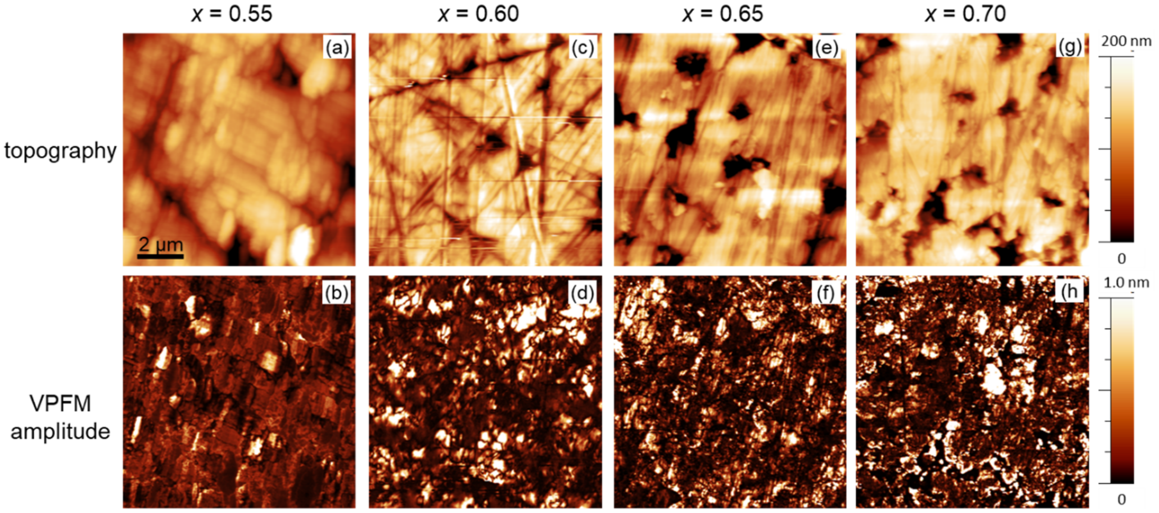

Figure S1. Topography and vertical PFM amplitude images of as-synthesized $(1\text{-}x)BiMg_{0.5}Ti_{0.5}O_3$-$xBiZn_{0.5}Ti_{0.5}O_3$ ceramics: x = 0.55 (a, b), 0.60 (c, d), 0.65 (e, f), 0.70 (g, h).

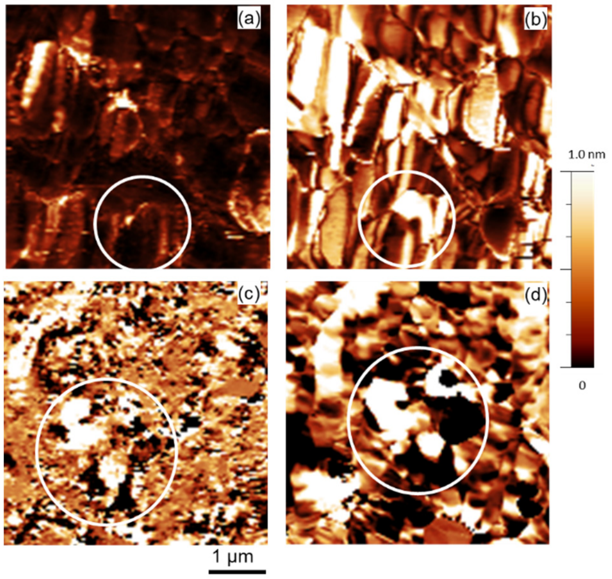

Figure S2. PFM amplitude images of as-synthesized $0.45BiMg_{0.5}Ti_{0.5}O_3$-$0.55BiZn_{0.5}Ti_{0.5}O_3$ (a, b) and $0.35BiMg_{0.5}Ti_{0.5}O_3$-$0.65BiZn_{0.5}Ti_{0.5}O_3$ (c, d) ceramics before (a, c) and after (b, d) annealing. White circles mark the same locations in the as-prepared and annealed ceramics.
